# Research on Intelligent Robot Point Cloud Grasping in Internet of Things

**DOI:** 10.3390/mi13111999

**Published:** 2022-11-17

**Authors:** Zhongyu Wang, Shaobo Li, Qiang Bai, Qisong Song, Xingxing Zhang, Ruiqiang Pu

**Affiliations:** 1 Key Laboratory of Advanced Manufacturing Technology of the Ministry of Education, Guizhou University, Guiyang 550025, China; 2 State Key Laboratory of Public Big Data, Guizhou University, Guiyang 550025, China; 3 School of Mechanical Engineering, Guiyang University, Guiyang 550025, China; 4 College of Mechanical Engineering, Guizhou University, Guiyang 550025, China

**Keywords:** object point cloud, 6-Dof grasp, attention mechanism, PointNet, robot, IoT

## Abstract

The development of Internet of Things (IoT) technology has enabled intelligent robots to have more sensing and decision-making capabilities, broadening the application areas of robots. Grasping operation is one of the basic tasks of intelligent robots, and vision-based robot grasping technology can enable robots to perform dexterous grasping. Compared with 2D images, 3D point clouds based on objects can generate more reasonable and stable grasping poses. In this paper, we propose a new algorithm structure based on the PointNet network to process object point cloud information. First, we use the T-Net network to align the point cloud to ensure its rotation invariance; then we use a multilayer perceptron to extract point cloud characteristics and use the symmetric function to get global features, while adding the point cloud characteristics attention mechanism to make the network more focused on the object local point cloud. Finally, a grasp quality evaluation network is proposed to evaluate the quality of the generated candidate grasp positions, and the grasp with the highest score is obtained. A grasping dataset is generated based on the YCB dataset to train the proposed network, which achieves excellent classification accuracy. The actual grasping experiments are carried out using the Baxter robot and compared with the existing methods; the proposed method achieves good grasping effect.

## 1. Introduction

The development of Internet of Things (IoT) technology has promoted the progress of a new generation of information technology [1]. With the deep integration of artificial intelligence with the Internet, IoT, big data and cloud platforms, as well as the progress of sensor technology for data collection and algorithms for data processing, IoT has made a significant breakthrough in the perception layer and network layer. With the support of super computing power, the application layer of IoT has also been developed rapidly, resulting in a large number of intelligent application products [2]. As a typical representative of the IoT application layer, intelligent robots benefit from the advancement of sensor technologies such as vision cameras and LIDAR and the development of deep learning algorithms, which can acquire more perception and decision-making capabilities and become more dexterous and versatile [3]. Letting robots imitate human-like flexibility to complete the grasping operation of unknown objects has been a hot issue in the field of robot grasping, and it is also a key area in the intersection of machine vision and robotics research [4]. In this paper, we investigate the problem of intelligent robot grasping and propose a robot grasping method based on the target point cloud to improve the grasping success rate of the robot.

Traditional robot autonomous grasping methods are based on form closure [5] or the force closure criterion [6] to plan the grasping pose, which requires acquiring the 3D model data of the target object in advance, and the grasping methods have low efficiency and poor adaptability for grasping objects with many dynamic changes and uncertainties. With the development of machine learning, deep learning technology represented by convolutional neural networks (CNNs) has made significant breakthroughs in several fields, mainly in computer vision, and has also promoted the development of vision-based robot grasping technology [7]. Most of the current grasping methods take 2D images or depth images as input, feature extraction of the input images by CNNs, and the final output of the grasped bit pose. Some researches used RGB images as input, first into a large number of candidate grasping frames, and then further optimized them to obtain the final grasping position [8,9,10]. The grasping method of Ref. [11] is similar to the above method, and the information of the depth channel of the image is added in the input stage to achieve more accurate grasping locations. Ref. [12] constructed a depth map-based grasp quality dataset and trained to obtain a grasp quality evaluation network, which first generates hundreds of candidate grasp locations on the depth map, then selects the highest quality candidate grasp location for grasping by the trained grasp quality evaluation network and thus is a two-stage grasping method. The above grasping method is probabilistically distributed for each object placement in the plane when the training data is generated. When extended to any angle, the data under many viewpoints do not exist in the training set, and the network may not be able to learn the position suitable for grasping. Therefore, the above method is suitable for grasping from a single angle in a fixed setting, and not from an arbitrary angle. To achieve arbitrary angle grasping, the grasping pose of the object 6-Dof needs to be acquired. Ref. [13] used the traditional method to generate the candidate grasp, and then used the depth images at three angles as input to perform the grasp quality estimation based on CNNs, and finally filtered the best location to achieve 6-Dof grasp. Ref. [14] also used traditional methods to screen candidate grasping positions, took the internal point cloud of the grasping device as input, used the PointNet [15] network to estimate the grasping quality, and finally output the best grasping position to complete grasping. Ref. [16] also took the candidate grasping point cloud as input, and used the PointNet++ [17] network to evaluate the grasping quality to obtain the best grasping and achieve better grasping effect. The above three methods can complete spatial 6-Dof grasping, among which Refs. [14,16] used point clouds as input. However, some traditional deep learning methods in the image domain cannot be directly applied to the point cloud domain due to the inherent unstructured and disordered characteristics of point cloud data. Therefore, these two methods used the PointNet and PointNet++ networks, the typical models for dealing with point clouds, to process point cloud data. Most of the existing methods to enhance the performance of point cloud networks enrich the input of the network or improve the feature extraction capability of the network. The attention mechanism can adaptively generate the weights of optimized network features to help the network learn what information needs to be emphasized or suppressed and extract features more precisely, which is ideal for extracting point cloud features.

**Figure 1:**
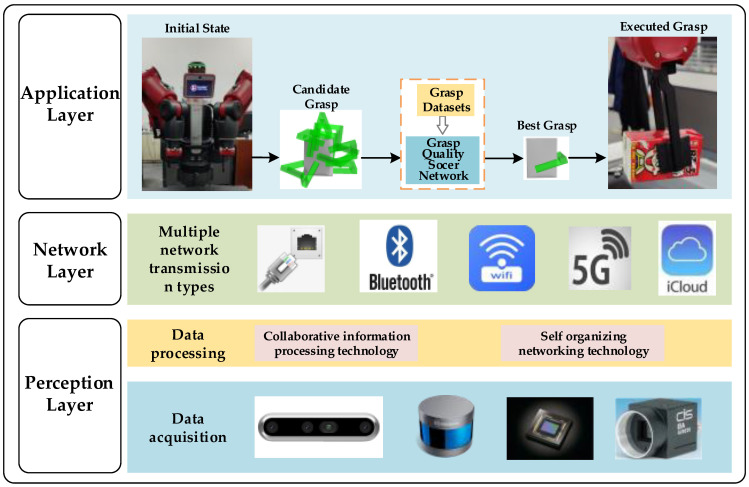
IoT architecture—Intelligent robot point cloud grasping.

The intelligent robot grasping system based on IoT includes the perception layer, the network layer and the execution layer, as shown in Figure 1, and this paper focuses on intelligent robot grasping as the application layer. Integrating the above analysis, this paper takes the object point cloud information as the input of the model, so that the grasping method can adapt to complex grasping scenes, while applying the attention mechanism to the point cloud processing network to improve the accuracy of the model. The actual grasping experiments show that the method has a high success rate and good generalization ability. The main contributions of our work can be summarized as follows:

(1) This study designs a grasping quality evaluation network based on the PointNet network, which is used to evaluate the quality of the generated candidate grasping positions, and a plug-and-play lightweight attention mechanism for point clouds that can be embedded in the feature extraction phase of the PointNet network to improve the network performance without significantly increasing the computational cost.
(2) Generating a grasp dataset containing object grasp location and quality labels based on the YCB dataset [18] for training our proposed grasp quality evaluation network.
(3) The actual grasping experiments are carried out with the Baxter robot and compared with the existing methods; the results show that our method has higher accuracy and higher grasping success rate.

The structure of this paper is arranged as follows: Section 2 introduces the processing method of point cloud data and the application of attention mechanism, and introduces the robot grasping method based on point clouds. Section 3 analyzes the processing of point cloud data by the PointNet network and the working principle of the attention mechanism. Section 4 proposes the grasping quality evaluation network. Section 5 trains and evaluates the network and conducts actual grasping experiments. Section 6 concludes our work and provides an outlook on future work.

## 2. Related Work

### 2.1. Processing of Point Cloud Data

In 3D space, each point can be represented as a vector, and the point cloud is a collection of these vectors. These vectors are usually expressed in the form of 3D coordinates (XYZ) in space and can be used to represent the shape of the object. Other elements can also be added after the position information to enrich the point cloud information, such as RGB color, gray value, category, etc. Compared with planar 2D images, 3D point cloud data have the following advantages:(1) It can express the geometric shape information and spatial position and attitude of objects more truly and accurately. (2) It is less affected by the change of illumination intensity, imaging distance and viewpoint. (3) There are no problems such as projection transformation in 2D images. Different from 2D image data, which can be represented as a matrix in the computer, point clouds are a kind of unstructured data. In terms of geometric features, the same group of point clouds can be represented as matrices of various permutations and combinations [19]. Traditional point cloud processing methods can be divided into two categories: One is to project the point cloud data onto a two-dimensional plane and process it according to some specific perspectives, and then combine the data from different perspectives to find the relationship between them, to understand the point cloud data, and the classical algorithms include MV3D [20] and AVOD [21]. The other is to divide the point cloud data into a voxel grid and process it with 3D convolution and other methods. The accuracy of such algorithms depends on the delicacy of the partition space, and the complexity of 3D convolution is very high; the classical algorithms are VoxelNet [22] and PointPillars [23]. The point cloud processing algorithm based on deep learning can directly extract 3D features based on the target point cloud and perform various cognitive tasks of the point cloud, such as point cloud classification, semantic segmentation and object detection, etc. The current classical algorithms are PointNet series of networks [15,17], and Graph convolution series of networks, for example Ref. [24].

### 2.2. Robot Grasping Based on Object Point Cloud

In recent years, with the rapid development of low-cost depth sensors and lidar, the detection and recognition technology of 3D objects is also developing. Radars, 3D scanners, depth cameras and other devices are used to acquire the image and depth information of objects, sense the objects in 3D space, and estimate the spatial position and attitude of objects, so as to provide information for the grasping task of the robotic arm [25]. Ref. [13] proposed the GPD algorithm, which first used traditional methods for screening candidate grasp locations, and then used CNN for feasibility estimation based on depth images from three angles to filter the optimal grasping strategy. Ref. [14] proposed the PointNetGPD, which also used the traditional method to filter the candidate grasp locations, but instead of using the depth map to evaluate the grasp quality, the point cloud inside the grasper is used to generate the optimal grasp using PointNet for grasp quality evaluation. In Ref. [16], a 6-Dof GraspNet was proposed to obtain multiple candidate grasps by a grasp sampling network using a 3D point cloud as input, and then the candidate grasps were evaluated by a Grasp Evaluator, while the estimated grasp results were further optimized to be closer to a reasonable grasp, further improving the grasp success rate. Ref. [26] improved the grasp success rate by rooting the full 6-Dof grasp pose and width in the observed point cloud and reducing the dimensionality of the grasp representation to 4-Dof.

### 2.3. Attention Mechanism in Computer Vision

The attention mechanism optimizes the model and makes more accurate judgments by assigning different weights to different attention parts of the model and extracting more important and critical information from them. Ref. [27] was the first to use the attention mechanism on RNN models for image classification tasks and achieved good classification results, providing a new direction for the application of the attention mechanism in computer vision. Ref. [28] proposed the channel attention mechanism SE-Net, which aims to model the interdependence between different feature channels in a display manner, to automatically obtain the importance of each feature channel by means of network learning, and finally to assign different weight coefficients to each channel to strengthen the important features and suppress the non-important features. Ref. [29] is an improved model based on SE-Net, which maintains excellent performance while focusing on reducing the complexity of the model. Ref. [30] proposed the spatial attention STK network, which makes the model adaptively focus on task-relevant regions in the image and find the regions in the image with the highest contribution to the task. The CBAM network [31] used a multi-angle pooling approach to generate adaptive attention weights to generate channel and spatial attention, and fuses channel and spatial attention in a serial manner to improve the network performance.

The attention mechanism applied to point cloud processing has also been of wide concern to researchers. Unlike Ref. [32], which requires the manual design of a weight, point cloud attention mechanisms can help the network to learn weights adaptively so that the network automatically focuses on important features and suppresses non-essential ones. By constructing a graph on the point cloud, and then extracting features on the graph, Ref. [24] established the graph structure of each point and its surrounding points, and introduced the attention mechanism to calculate the edge weight of the center point and each adjacent point, so that the network can achieve better results in the segmented edge parts. Ref. [33] proposed an offset attention algorithm with an implicit Laplace operator and normalized optimization, which is displacement-invariant and more suitable for point cloud learning than the original self-attention module in Transformer, achieving advanced performance on tasks such as shape classification, partial segmentation and semantic segmentation.

## 3. Principal Analysis

The PointNet network mainly solves how to process 3D point clouds directly with 2D CNNs, which can extract point cloud features stably even if the point clouds are fluctuating, noisy or missing. In neural networks, the attention mechanism is usually an additional network that can autonomously select certain parts of the input or assign different weights to different parts of the input to filter out the important information from a large amount of information. This section analyzes the feature extraction process of PointNet networks and the principle of the attention mechanism.

### 3.1. PointNet Network Structure Analysis

**Figure 2:**
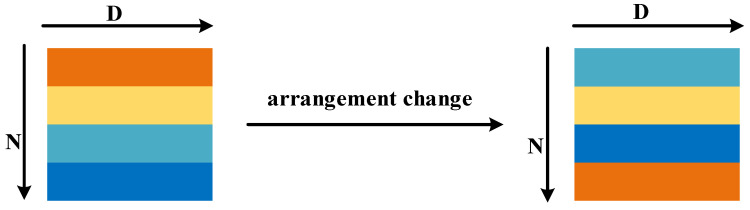
Permutation invariance of point clouds.

Point cloud data are unordered data; the order between points can be transformed arbitrarily, but they still represent the same object. As shown in Figure 2, when the input point cloud is D×N data, the model needs to be invariant to N permutations and the dimension of N is randomly scrambled; it should still represent the same object. This feature is usually realized by symmetric functions, such as Sum and Max.

(1) $$f(x_{1},x_{2},\ldots ,x_{n})=\max\{x_{1},x_{2},\ldots ,x_{n}\}$$

(2) $$f(x_{1},x_{2},\ldots ,x_{n})=x_{1}+x_{2}+\ldots +x_{n}$$

**Figure 3:**
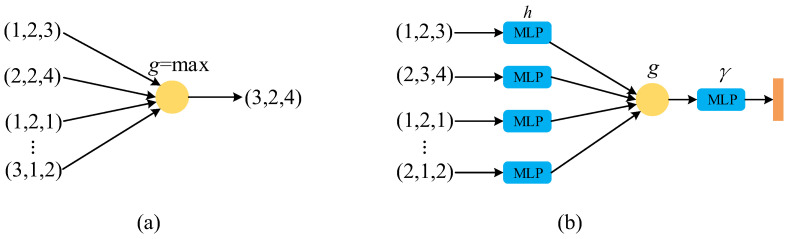
Simple feature extraction network. (**a**) Feature extraction using Max function; (**b**) PointNet network basic structure.

Therefore, the Max function can be used to design a simple feature extraction network initially, as shown in Figure 3a. The input is a set of N×3 point cloud data, where g=max means taking the maximum value of each dimensional feature, and the output is 1×3 data after completing feature extraction. Obviously, changing the arrangement order of the point cloud data has no effect on the output result.

However, the Max function only inherits the maximum feature value of each of the three dimensions in the feature extraction process. For a single point, too many features are lost, which will inevitably lead to partial information loss. To solve this problem, a clever solution is to map each point to a higher dimensional space before feature extraction with the Max function, so as not to lose too much information during feature extraction.

As shown in Figure 3b, h represents that each point is mapped to a redundant high-dimensional space, and then the symmetric function g is used for feature extraction. In this process, the feature loss of each point will be greatly reduced. Based on this, a prototype of PointNet can be designed, as shown in the Formula (3):

(3) $$f(x_{1},x_{2},\ldots ,x_{n})=\gamma *g(h(x_{1}),\ldots ,h(x_{n}))$$

where x represents a point in the input point cloud, h represents high-dimensional mapping for each point, g is a symmetric function representing feature extraction of higher dimensions, and finally a softmax classification is connected to form a basic PointNet network.

**Figure 4:**
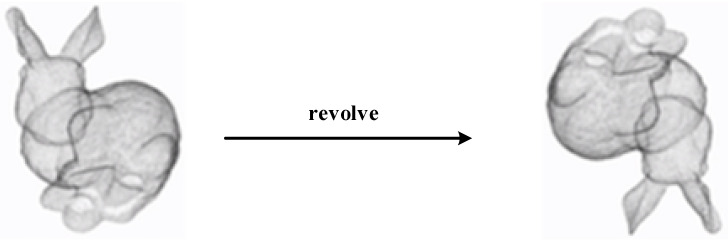
Rotation of an object.

Like 2D images, point cloud data do not change the shape characteristics of the object they represent with operations such as rotation and translation. As shown in Figure 4, the rotation invariance of a point cloud means that given an object, rotating its point cloud data by an angle will also change its x,y,z coordinates, but the representation is still the same object.

**Figure 5:**
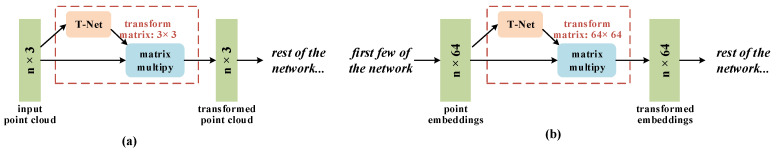
Point Cloud T-Net Network. (**a**) Input point cloud T-Net network; (**b**) Feature transformation T-Net network.

For the point cloud processing model, the network should be able to identify the same object point cloud quickly and accurately no matter what angle it is presented at or in different coordinate systems. Therefore, before feature extraction, point cloud data should be aligned to ensure invariance. The rotation of the point cloud is very simple; it just needs multiplying an N×D point cloud matrix by a D×D rotation matrix. As shown in Figure 5a, the network input is N×3 point cloud data, and a 3×3 transformation matrix is obtained by a T-Net network, which is multiplied with the input matrix to obtain the rotation-transformed matrix, thus completing the correction of the input point cloud.

The input point cloud is upgraded to 64 dimensions after one feature extraction and then multiplied by a rotation matrix of 64×64 obtained through a T-Net network to transform the point cloud at the feature level, as shown in Figure 5b.

**Figure 6:**
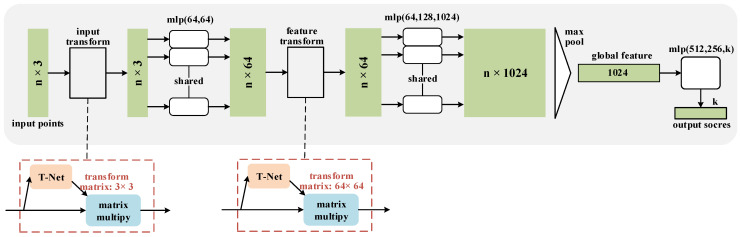
Overall structure of point cloud classification network. The dotted boxes are input point cloud T-Net network and feature transformation T-Net network respectively.

The overall structure of the point cloud classification network is shown in Figure 6 [15]. For each N×3 point cloud input, the network first aligns it in space by input transform, then performs feature extraction using a Multi-layer Perceptron (MLP) to map it to 64-dimensional space, then aligns it in feature dimension by feature transform, performs feature extraction using the MLP and finally maps it to 1024-dimensional space. At this time, each point of the point cloud is a 1024-dimensional feature vector. The maximum pooling is introduced as a symmetric function to obtain the 1×1024 global features of the point cloud, and then a fully connected cascade network is connected to achieve a k classification result.

### 3.2. Analysis of Attention Mechanism in Computer Vision

There are various ways to introduce attention mechanisms in neural networks, and in the case of CNNs, for example, attention mechanisms can be introduced in the spatial dimension [30], or in the channel dimension [28], or in a mixture of spatial and channel dimensions [31].

**Figure 7:**
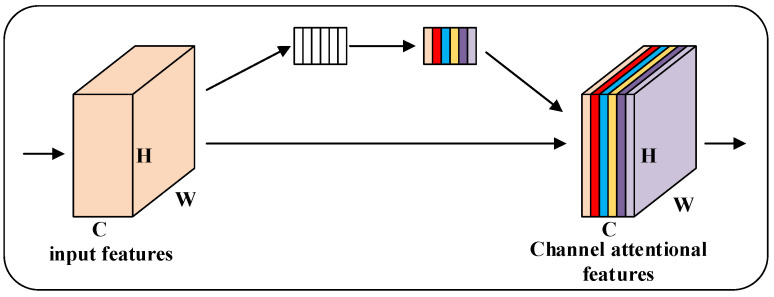
Channel attention mechanism.

The channel attention mechanism uses a new neural network to obtain the importance of each channel of the feature graph by automatic learning and then uses this importance to assign a weight value to each feature so that the neural network focuses on certain feature channels, boosts the channels of the feature graph that are useful for the current task and suppresses the feature channels that are not very useful for the current task. As shown in Figure 7, before the input channel attention mechanism, the importance of each channel of the feature map is the same, and through the channel attention mechanism, the importance of each feature channel becomes different; different colors represent different weights, so that the neural network focuses on certain channels with large weight values.

First, the feature map is compressed in spatial dimensions by global average pooling, and the dimensions are compressed from [C,H,W] to [C,1,1]; then weights are generated for each feature channel by the MLP network with shared weights, which represents the influence of each channel on feature extraction, and this weight is applied to each of the original feature channels, i.e., each channel is multiplied by its respective weight, and the importance of each channel can be learned. The channel attention mechanism can be represented by Formula (4):

(4) $$\begin{array}{l} M_{c}\left(F\right)=\sigma (\mathrm{MLP}(AvgPool(F))+\mathrm{MLP}(MaxPool(F)))\\ =\sigma (W_{1}(W_{0}(F_{avg}^{c}))+W_{1}(W_{0}(F_{max}^{c}))) \end{array}$$

where $F_{avg}^{s}$ and $F_{\max}^{s}$ are the feature expressions for the average pooling and maximum pooling, respectively.

**Figure 8:**
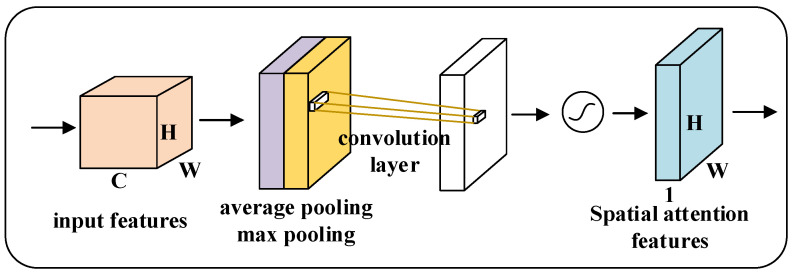
Spatial attention mechanism.

The spatial attention mechanism is used to distinguish the degree of contribution of different regions in the image to the task, as shown in Figure 8. First, average pooling and maximum pooling are performed in the channel dimension to compress the channels, respectively, to obtain two feature maps of dimension [1,H,W]. Then, these two feature maps are stacked in the channel dimension to become a feature map of dimension [2,H,W], and the feature map dimension is changed to [1,H,W] by fusing the channel information using a 7×7 (or 3×3) size convolution kernel. Finally, the spatial weights of the feature map are normalized by the sigmoid function to obtain the weights of different regions, and then the importance of different regions to the task can be obtained by multiplying the input feature map and the weights. The calculation process of spatial attention mechanism is shown in Formula (5):

(5) $$\begin{array}{ll} M_{s}\left(F\right) & =\sigma (f^{7\times 7}([AvgPool(F);MaxPool(F)]))\\ & =\sigma (f^{7\times 7}([F_{avg}^{s};F_{max}^{s}])) \end{array}$$

**Figure 9:**
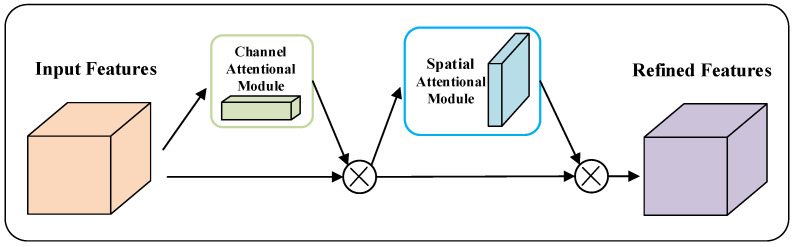
Convergence channel and spatial attention mechanism.

Spatial attention allows the neural network to pay more attention to the regions that are decisive for the task and ignore irrelevant regions, while channel attention is used to deal with the assignment relationship of feature map channels. Combining spatial attention and channel attention into one network and simultaneously assigning attention to both dimensions enhances the effect of the attention mechanism on model performance, as shown in Figure 9.

## 4. Grasping Quality Classification Network Incorporating Attention Mechanism

Given the excellent point cloud classification performance, PointNet can be applied to evaluate grasping performance. The point cloud attention mechanism is added to make the network better extract the local point cloud that the classification task focuses on and to improve the network’s accuracy. This section describes the specific structure of the point cloud classification network for grasp quality evaluation, designs the point cloud attention mechanism and finally proposes the PointNet grasp quality classification network incorporating the attention mechanism.

### 4.1. Structure Design of Point Cloud Classification Network

**Figure 10:**
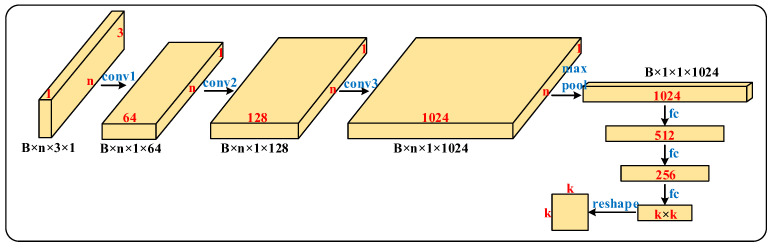
T-Net network structure.

Based on the analysis in Section 3.1, before feature extraction, PointNet learns a 3×3 transformation matrix through the T-Net network, multiplies it with the input point cloud and performs alignment operations on the input point cloud. The structure of the T-Net network is shown in Figure 10. The input point cloud data are treated as an n×3×1 single-channel image, and after three times of convolution and one pooling, reshaping is to 1024 nodes, then two fully connected layers are connected and finally the output is reconstructed into a k×k matrix. The ReLU activation function and batch normalization are used for all but the last layer of the network. For the input transform, k=3.

The input point cloud is upgraded to 64 dimensions after one feature extraction, and a 64×64 rotation matrix is learned by the T-Net network, which is multiplied with the 64-dimensional point cloud to transform the point cloud at the feature level. As a feature transform, the T-Net network has k=64. Since it is difficult to optimize the high-dimensional space, a regularization penalty term needs to be introduced to reduce the difficulty of optimization, as shown in the Formula (6).

(6) $$L_{reg}={\left\|I-AA^{T}\right\|}_{F}^{2}$$

where A is a k×k dimensional transformation matrix obtained from the T-Net network learning, and the regularization term makes A close to an orthogonal matrix.

**Figure 11:**
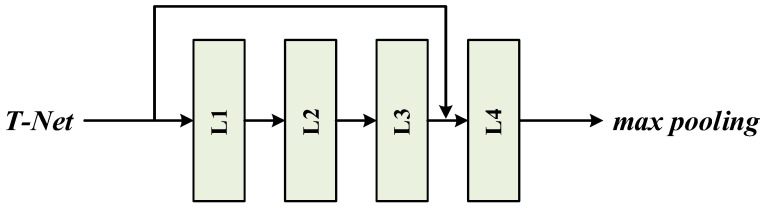
Skip connection layer.

The MLP network is used to extract features from the input point cloud [15]. Drawing on the idea of a residual network [34], by connecting the features of different layers, the low-level features and the high-level features are fused to realize the feature reuse and make full use of the features of different levels, as shown in Figure 11. Adding a skip connection layer makes the transfer of features and gradients more efficient and makes the network training simpler.

Each layer of the MLP network is subjected to batch normalization and uses ReLU as the activation function. The MLP network can extract the M-dimensional features of each point and then obtain the 1024-dimensional features of the point cloud through the maximum pooling operation.

The grasp quality evaluation network finally completes the quality classification of the input candidate grasp, using cross entropy as the loss function. Kullback-Leibler(KL) divergence can be used to measure the difference between the true distribution P(x) of the sample and the distribution Q(x) predicted by the model, as shown in Formula (7):

(7) $$D_{KL}(p∥q)=\sum_{i=1}^{n}p(x_{i})\log\left(\frac{p(x_{i})}{q(x_{i})}\right)$$

where P(x) represents the true distribution of the sample, Q(x) represents the distribution predicted by the model and n represents all possibilities of the event. The smaller the KL value, the closer the distribution of P(x) and Q(x), and Q(x) can be trained repeatedly to make its distribution approximate P(x).

Taking apart the KL divergence formula, Formula (8) is obtained as follows:

(8) $$\begin{array}{ll} D_{KL}(p∥q) & =\sum_{i=1}^{n}p(x_{i})\log(p(x_{i}))-\sum_{i=1}^{n}p(x_{i})\log(q(x_{i}))\\ & =-H(p(x_{i}))+\left[-\sum_{i=1}^{n}p(x_{i})\log(q(x_{i}))\right] \end{array}$$

where $-H(p(x_{i}))$ represents the information entropy of $x_{i}$. Since our model is trained as supervised training, the labels of the samples have been determined, that is, the true distribution P(x) of the samples is known, so $-H(p(x_{i}))$ is a fixed value. Based on Formula (8), the cross entropy can be obtained as shown in Formula (9):

(9) $$H(p,q)=-\sum_{i=1}^{n}p(x_{i})\log(q(x_{i}))$$

The softmax function is used to map the model output to the interval (0,1), and the results of multiple classification are presented in the form of probability, as shown in Formula (10):

(10) $$S_{j}=\frac{e^{Z_{j}}}{\sum_{i=1}^{k}e^{Z_{k}}}$$

where $z_{j}$ and $S_{j}$ represent the input and output of the j−th neuron, respectively. The cross-entropy loss function is obtained by substituting $S_{j}$ into the cross-entropy, as shown in Formula (11):

(11) $$L=-\sum_{i=1}^{n}y_{i}\log S_{j}$$

where $y_{i}$ represents the sample label.

### 4.2. Point Cloud Attention Mechanism Network Design

Based on the theoretical analysis in Section 3.2, similar to the application of attention mechanisms in the image processing domain, we designed two point cloud attention mechanisms by pooling along the feature channel number C dimension and the point cloud number N dimension, respectively.

(12) $$P_{c}=A_{c}(P)⊗P$$

(13) $$P_{n}=A_{n}(P)⊗P$$

where $P\in {\mathbb{R}}^{B\times N\times 1\times C}$ denotes point cloud data (B,N,C denote batch size, number of points and number of feature channels, respectively), $A_{c}\in {\mathbb{R}}^{B\times N\times 1\times C}$ denotes point cloud feature attention mechanism, $P_{c}$ denotes output features of point cloud feature attention mechanism, $A_{n}\in {\mathbb{R}}^{B\times N\times 1\times C}$ denotes point cloud channel attention mechanism, $P_{n}$ denotes output features of point cloud channel attention mechanism and ⊗ denotes matrix fork multiplication.

**Figure 12:**
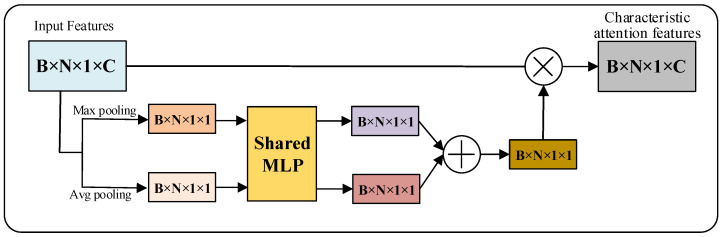
Point cloud feature attention mechanism $A_{c}$.

Different pooling methods are used to collect feature information with reference to the CBAM design approach in the image domain. First, feature aggregation is performed along the feature channel number C dimension using parallel average pooling and maximum pooling for the point cloud input features P to generate feature representations $P_{avg}^{c}$ and $P_{\max}^{c}$ from different angles. Then, the feature channel number dimension of the aggregated features is trained using a single hidden layer MLP network with shared parameters, which is used to generate attention weights. Finally, the sigmoid activation function is used to activate the weights. As shown in Formula (14):

(14) $$\begin{array}{ll} P_{c} & =\sigma \left(MLP(AvgPool(P))+MLP(MaxPool(P))\right)\\ & =\sigma (W(P_{\mathrm{avg}}^{c})+W(P_{\max}^{c})) \end{array}$$

where σ represents the Sigmoid activation function and W represents the weight of the MLP. The calculation process of the point cloud feature attention mechanism $A_{c}$ is shown in Figure 12.

**Figure 13:**
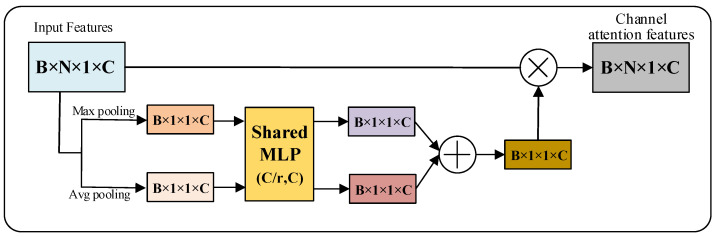
Point cloud channel attention mechanism $A_{n}$.

Similar to the point cloud feature attention mechanism, first, feature aggregation is performed on the point cloud input features P using parallel mean pooling and maximum pooling along the number N dimensions of the point cloud to generate feature representations $P_{avg}^{n}$ and $P_{\max}^{n}$ from different angles. Then, the aggregated features are trained using a double hidden layer MLP with shared parameters, and the point cloud feature channels C are first reduced and then restored with a reduction factor r, which is used to generate attention weights. Finally, the sigmoid activation function is used to activate the weights. As shown in Formula (15):

(15) $$\begin{array}{ll} P_{n} & =\sigma \left(MLP(AvgPool(P))+MLP(MaxPool(P))\right)\\ & =\sigma (W_{1}(W_{0}(P_{\mathrm{avg}}^{n}))+W_{1}(W_{0}(P_{\max}^{n}))) \end{array}$$

where σ represents the Sigmoid activation function, and $W_{0}$ and $W_{1}$ represent the weights of the MLP. The calculation process of the point cloud channel attention mechanism $A_{n}$ is shown in Figure 13.

**Figure 14:**
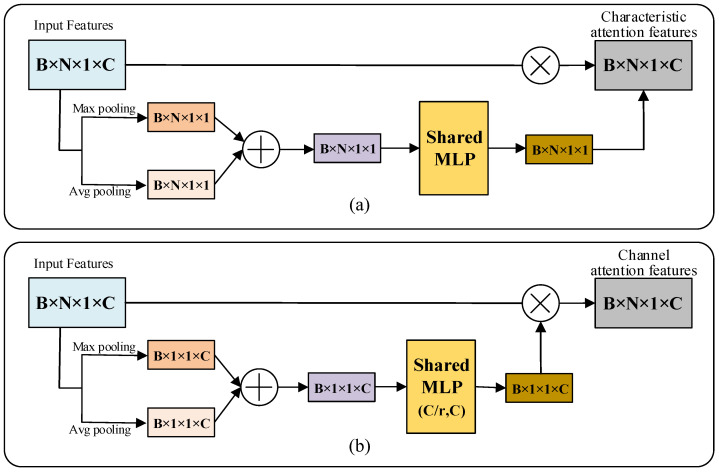
Point cloud attention mechanisms. (**a**) Point cloud feature attention mechanism $A_{c}{}^{*}$ for fusion first and training later; (**b**) Point cloud channel attention mechanism $A_{n}{}^{*}$ for fusion first and training later.

To investigate the effect of the fusion order of pooled features from multiple perspectives on network performance, we designed the attention mechanisms $A_{c}$ and $A_{n}$ for training first and then fusion, and the attention mechanisms $A_{c}{}^{*}$ (Figure 14a) and $A_{n}{}^{*}$ (Figure 14b) for fusion first and then training.

**Figure 15:**
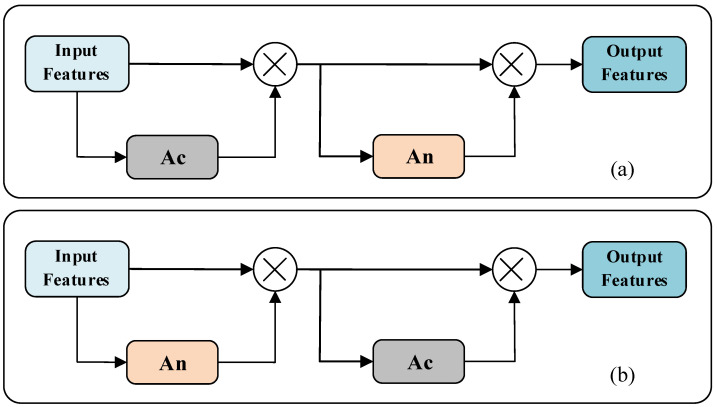
Converged point cloud attention mechanisms $A_{cn}$ and $A_{nc}$. (**a**) Converge $A_{c}$ first and then converge $A_{n}$; (**b**) Converge $A_{n}$ first and then converge $A_{c}$.

In order to study the impact on network performance when the feature attention mechanism and channel attention mechanism are used simultaneously, inspired by CBAM [31], the two attention mechanisms are fused and two schemes, $A_{cn}$ (Figure 15a) and $A_{nc}$ (Figure 15b), are designed according to the order in which the two attention mechanisms are used.

### 4.3. Design of PointNet Grasping Quality Classification Network Incorporating Attention Mechanism

**Figure 16:**
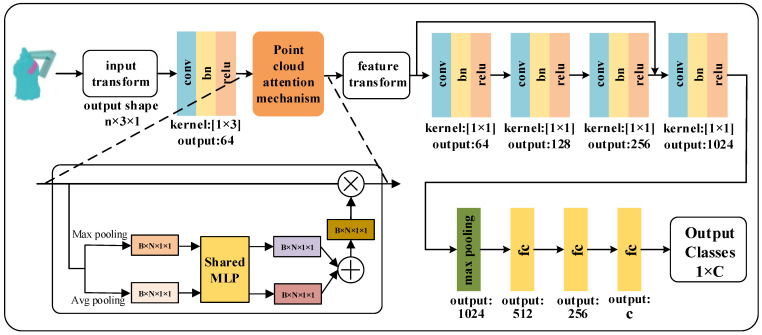
Overall structure of PointNet grasping quality classification network incorporating attention mechanism.

The point cloud attention mechanism we designed is embedded into the feature extraction stage of the PointNet network to make the network pay more attention to the local point clouds of the candidate grasping positions, and the PointNet grasping quality classification network incorporating the attention mechanism is designed, as shown in Figure 16. The network takes the original point cloud of the grasped poses as input, and through feature extraction and maximum pooling operations, the global features of the point cloud are obtained, and finally the quality level of the input grasped poses is classified.

## 5. Model Training and Actual Grasping Experiments

Firstly, a grasping dataset for model training is generated, then the rationality of our designed network is verified, the effectiveness of T-Net and the point cloud attention mechanism is investigated, and the optimal network structure is obtained. The actual grasping experiments were conducted and excellent crawling results were obtained.

### 5.1. Generating Grasping Dataset

Assuming that the object to be grasped is a rigid object, its initial state is defined as $I=(G_{0},D_{0})$, where $G_{0},D_{0}$ represent the geometric state and spatial position of the object, respectively. Assume that the contact mode between the object and the gripper is frictional contact. In this paper, only the two-finger gripper is considered, and the spatial position of the gripper is defined as $g=(s,r)\subset {\mathbb{R}}^{6}$, where s=(x,y,z), $r=(r_{x},r_{y},r_{z})$ represents the center position and angle of the gripper, respectively, and a candidate grasping position can be expressed as G=(I,g).

#### 5.1.1. Sampling of Candidate Grasp Positions

A total of 77 common objects in the YCB dataset [18] are selected, and their initial geometric states and spatial locations are known. Random sampling is performed on the object model grid to form a series of symmetric points $c_{i1}$, $c_{i2}$ on the two surfaces of the model. The gripper angle is limited to (−π/2,π/2). Under the condition that the initial state of the object is known, a set of candidate grasping positions can be expressed as $G=((c_{i1}+c_{i2})/2,r)$. The gripping positions where the gripper and the object may collide are eliminated from the generated set of candidate gripping positions, and the remaining candidate gripping positions are further filtered according to the force closure criterion, and the candidate gripping positions with force closure are finally retained.

#### 5.1.2. Generating Training Labels

According to the force closure criterion [35], the quality of the candidate grasp position is evaluated [36], and the binary label can be obtained, i.e., 0 for a failed grasp and 1 for a successful grasp. Based on this, a series of labeled grasp candidates can be obtained for a given friction coefficient. In order to generate more candidate grasps and further obtain the best candidate grasp, we increase the friction coefficient in the range of [0.1, 1.0] by 0.1 each time to determine whether the generated candidate grasp is force-closed. Generally, better grasps tend to require less friction, so let λ=1/μ denote the quality score of candidate grasps, and the smaller μ is, the larger λ is and the higher the quality score of grasps.

First, up to 50 sets of candidate grasping positions G are randomly generated for each object on its surface, and up to 20 sets of valid candidate grasping positions (i.e., no collision between the gripper and the object) are retained after initial screening. Within the range [0.1, 1.0], we use different friction coefficients to evaluate the quality of the retained candidate grasping positions and generate candidate grasps $G=((c_{i1}+c_{i2})/2,r,\lambda )$ with quality labels. The input of the grasp quality evaluation model is point cloud data, so the model of each object and the generated grasps are converted to point cloud data by the ICP algorithm to finally obtain the grasping dataset.

### 5.2. Training the Generated Network

We divided the friction coefficient μ into 5 equal parts in the range of [0.1,1.0], and then the corresponding grasping quality λ was also divided into 5 classes; therefore, our model was set as a five-class model. We divided the generated grasp dataset into training set and test set according to the ratio of 4:1, which were used for training and testing of the model, respectively. The Adam optimizer was selected to optimize the whole network, and the initial learning rate was set as 0.005, and then the learning rate was updated according to the Formula (16):

(16) $$\alpha =\alpha_{0}\times \gamma^{\left\lfloor epoch/step\right\rfloor }$$

where α is the current learning rate, $\alpha_{0}$ is the initial value of the learning rate, epoch is the number of iteration steps of the current training, step is the period of learning rate decline, γ is the decline factor and ⌊epoch/step⌋ represents the downward rounded value of epoch/step. We set $\alpha_{0}=0.005$, γ=0.9, step=30 and trained 200 epochs. The training environment is Ubuntu18.04 64-bit OS, using PyTorch deep learning framework, hardware configuration: Intel I9-9900X, RAM 128GB, NVIDIA GeForce RTX2080Ti*2.

### 5.3. Effect of Verification Module on Network Accuracy

In order to verify the effectiveness of the input transform and feature transform modules added to the network, we set up the following comparative experiment, as shown in the Table 1. On the premise of ensuring the reasonable overall structure of the network, the influence of the two modules on the network’s accuracy is verified, respectively.

As can be seen from the Table 1, when the transform module is not used, the network accuracy decreases by about 2.76%, while when the input transform module is used alone, the improvement of network accuracy is very limited. At the same time, it is noted that due to the large dimension of the feature transform module, the network accuracy will decrease when regularization is not added. When feature transform and regularization are used together, the network accuracy will be significantly improved. When two modules are used at the same time and regularization is added, the network accuracy is relatively improved.

In order to verify the effect of different attention mechanisms on model accuracy, we set up comparison experiments as shown in Table 2.

The point cloud feature attention mechanism $A_{c}$ and the point cloud channel attention mechanism $A_{n}$ improve the model accuracy to 91.30% and 90.62%, respectively, which are 1.59% and 0.91% higher than the original model, respectively. The results show that both point cloud attention mechanisms designed in this paper play a positive role in feature extraction of point cloud data, which verifies the rationality of the attention mechanism design, and the point cloud feature attention mechanism $A_{c}$ has a better effect. The accuracy of the attention mechanisms $A_{c}{}^{*}$ and $A_{n}{}^{*}$, which are fused first and trained later, is improved compared with the original network, but the improvement effect is not as good as that of the attention mechanisms $A_{c}$ and $A_{n}$, which are trained first and fused later, indicating that the strategy of fusing first and training later leads to some information loss in the fusion process of multi-angle features, and thus the improvement effect on the model accuracy is poor. It is also noted that, unlike the experience of CBAM in image attention mechanisms, the design solutions $A_{cn}$ and $A_{nc}$, which fuse two attention mechanisms, do not further improve the model accuracy. Therefore, we finally chose $A_{c}$ as the point cloud attention mechanism.

In order to investigate the best position of the attention mechanism to be used in the model, we set up the following comparison experiments: I, II, III, IV, V, VI denote the embedded attention mechanism $A_{c}$ at model feature dimensions of 3, 64, 64, 64, 128, 1024, respectively.

As shown in Table 3, when the attention mechanism $A_{c}$ is used directly after layer I, the accuracy improves compared to the original network. When the attention mechanism is used after layers II and III, the accuracy is 91.30% and 90.89%, respectively, which is a large improvement compared to the original network, while the accuracy improves less or even decreases when the attention mechanism continues to be used at a deeper level. The results show that our designed attention mechanism for point clouds is more suitable for embedding in the shallow layers of the network.

GPD [13] is an advanced work for 6-Dof grasp detection, which first completes the sampling of grasping bit poses by the Darboux framework, then evaluates its quality by using the trained grasping quality evaluation model and finally completes the grasping. Therefore, we chose GPD (3 channels and 12 channels) as our baseline method, generated the training dataset according to its grasp sampling strategy, and then trained the model and compared it with the related grasping methods proposed in recent years.

As shown in Table 4, the classification accuracy of our model can reach 91.30%, which is 11.59% higher than GPD (3 channels), 4.96% higher than GPD (12 channels), 4.19% higher than S^4^G, and 1.05% higher than Contact-GraspNet, indicating that our model has good classification of grasping quality performance.

### 5.4. Actual Grasping Experiments

**Figure 17:**
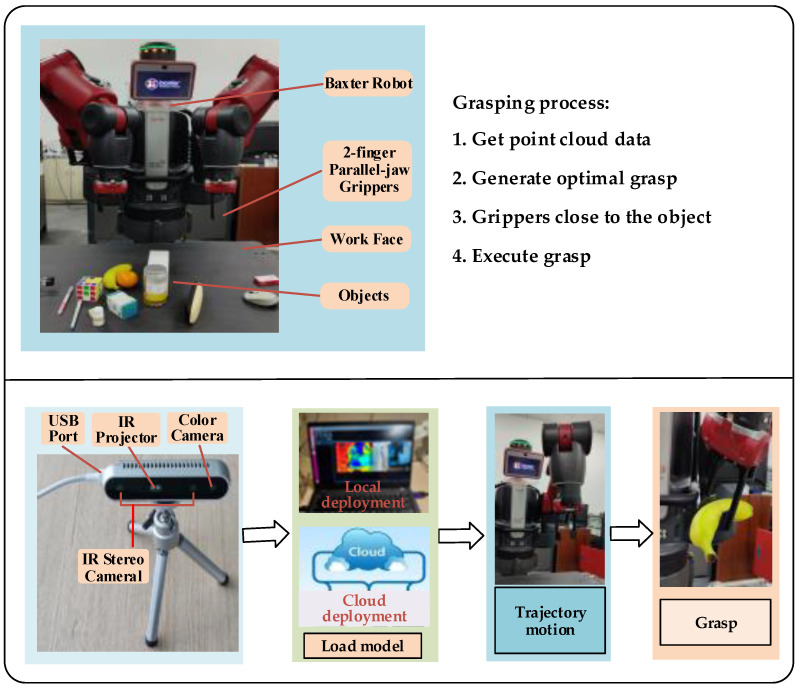
Grasping experiment frame.

Robotic grasping in IoT, which contains the sensing layer, the network layer and the application layer, involves a series of operations such as point cloud information collection, model loading, trajectory planning and performing grasping. The experimental framework of grasping in this paper is shown in Figure 17, and the whole system is controlled by the ROS platform. First, the Intel RealSense D415 depth camera is used as the perception module to obtain the point cloud. Then the information is uploaded to the ROS platform for processing, and the trained model can be deployed on the local server or on the cloud server and loaded through the network. Finally, the Baxter robot is used to execute the grasping operation.

**Figure 18:**
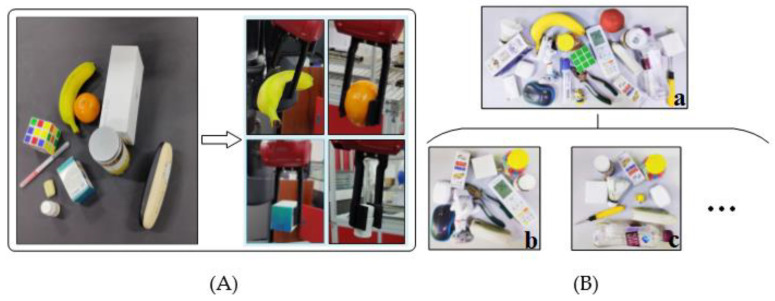
Grasping experiment. (**A**)Single-object grasping; (**B**) Cluttered grasping scene.

Ten common objects were selected as candidate grasping objects for the single-object grasping experiment, as shown in Figure 18A, and each object was placed on the working surface in turn, with the initial position and attitude of the object randomized at each placement. A successful grasp is defined as: (1) the two-finger parallel gripper successfully grabs the object; (2) the object is moved horizontally for a distance of 30cm without the object falling. Each grasping time is limited to 60 s, and if the grasping is not completed after 60 s, it is considered as a failure. Each object was grasped 20 times, and the success rate of grasping was calculated. The experimental results are shown in Table 5.

As can be seen from Table 5, the grasping success rate of our model for single objects can reach 93.50%, which is 12.00% higher than that of the GPD model, indicating that our proposed method can well plan and execute grasping. At the same time, we find that the average success rate of all models for single objects is above 80%. The analysis reasons are as follows: Firstly, because the 10 objects captured are all simple and relatively regular objects, the model can generate more high-quality candidate grasps on their surfaces. Secondly, these 10 objects are all included in the object types that constitute the data set, so the success rate of grasping by several methods is higher.

To further verify the generalization ability of the model, we constructed a grasping object set consisting of 20 objects of different shapes and masses (as shown in Figure 18(B-a)), including 10 objects that did not appear in the training dataset, such as doll model, pliers, badminton, etc. We defined a round of grasping experiments as follows: (1) 10 objects are randomly selected from the grasping object set; (2) these 10 objects are placed on the working surface at the same time, and the position and pose of each object are random, forming a cluttered grasping scene (as shown in Figure 18(B-b,B-c)); (3) the objects are sorted out from the cluttered scene in turn using the two-finger parallel jaws, and multiple grasping is performed until all objects are sorted or the specified number of grasps n is reached. Ten rounds of experiments were conducted for each model, and the number of grasps per round was set to n=15. We used the success rate and completion rate as the quality evaluation metrics of the model, where the success rate represents the average grasping accuracy of each object, and the completion rate represents the percentage of objects successfully removed from the cluttered scene after n grasps are performed. The experimental results are shown in Table 6.

The method in this paper uses the object point cloud as the information input, which improves the success rate of grasping in cluttered scenes, and introduces the point cloud attention mechanism, which enables the network to focus on the point cloud information in the closed area of the grasper and further improves the efficiency of the model. Therefore, the proposed method in this paper has better experimental results. From Table 6, we can see that our model has a higher grasping success rate and completion rate in cluttered scenes compared to the baseline; especially, the completion rate is 13.00% higher than GPD (3 channels). The grasping success rate of our model is 81.00%, while the grasping success rate of the four models is all lower than that of the single-object grasping. The following factors contribute to this result: (1) when multiple objects are placed together, there is a possibility that the objects may block each other, which affects the camera’s ability to capture the complete outline of the target object; (2) the point cloud captured for transparent objects tends to be sparse, while the friction coefficient is difficult to determine for smooth objects, which cannot form an effective grasp.

## 6. Conclusions and Future Work

The continuous development of IoT technology has broadened the application areas of robots, and the advancement of technologies such as vision sensors and computer vision has enabled intelligent robots to perform various tasks dexterously. In this paper, we address the problem of vision-based intelligent robot grasping and propose a PointNet-based grasping quality evaluation network to process point cloud information and classify the quality of the generated candidate grasps. Through comparative experiments, the impact of the two T-Net networks and the point cloud attention mechanism on the overall accuracy is verified, and it is found that the model accuracy is substantially improved when input transform, feature transform and regular term are used simultaneously. When the attention mechanism $A_{c}$ pools the point cloud features along the feature channel number dimension and learns features with attention weighting by MLP, it can further enrich the point cloud feature information on the base network and the network performance improvement is more obvious, which shows that the unstructured point cloud data with only (x,y,z) coordinate information and its single feature information are still a problem that point cloud feature learning must focus on. We selected common objects in daily life and used a Baxter robot to carry out the actual grasping experiments, including the single-object grasping experiment and cluttered scene grasping experiment. Compared with the existing grasping methods, our method has higher accuracy, especially in cluttered scenes, and the grasping success rate and completion rate reached 81.00% and 95.00%, respectively.

Real-life grasping scenes are often complex scenes with noise and occlusion, and the point cloud information from a single viewpoint sometimes cannot contain the complete object surface contour, which may fail to generate higher quality candidate grasps and lead to a decrease in the grasping success rate. We will consider information acquisition of objects through multiple viewpoints to generate better grasping poses and further improve the grasping success rate in cluttered scenes, thus opening up more application scenarios of intelligent robots in IoT.

## Figures and Tables

**Table 1:** Effect of T-Net network on model accuracy.

Model	Module	Accuracy
PointNet (vanilla)	none	86.95%
PointNet	input	87.59%
PointNet	feature	86.77%
PointNet	feature + reg.	88.12%
PointNet	both	89.71%

**Table 2:** Effect of attentional mechanisms on model accuracy.

Model	AM	Accuracy
PointNet	-	89.71%
PointNet	$A_{c}$	91.30%
PointNet	$A_{c}{}^{*}$	90.41%
PointNet	$A_{n}$	90.62%
PointNet	$A_{n}{}^{*}$	90.13%
PointNet	$A_{cn}$	89.53%
PointNet	$A_{nc}$	89.32%

**Table 3:** Comparison of where attentional mechanisms are used in the model.

Model	Location	Accuracy
PointNet	-	89.71%
PointNet	I	90.34%
PointNet	II	91.30%
PointNet	III	90.89%
PointNet	IV	90.64%
PointNet	V	89.25%
PointNet	VI	88.92%

**Table 4:** Model training results.

Method	Input Data	Year	Accuracy
GPD (3 channels) [13]	point cloud	2017	79.71%
GPD (12 channels) [13]	point cloud	2017	86.34%
S^4^G [37]	point cloud	2019	87.11%
Contact-GraspNet [26]	point cloud	2021	90.25%
Ours	point cloud	2022	91.30%

**Table 5:** Experimental results of single-object grasping.

Method	Banana	Glasses-Case	Medicine Bottle	Packing Box	Orange	Rubber	Rubik’s Cube	Pen	Medicine Box	Tea Bottle	Average
GPD (3 channels)	85.00%	80.00%	85.00%	80.00%	85.00%	75.00%	90.00%	75.00%	80.00%	80.00%	81.50%
GPD (12 channels)	95.00%	80.00%	85.00%	80.00%	90.00%	80.00%	95.00%	85.00%	90.00%	90.00%	87.00%
S^4^G	100.00%	85.00%	85.00%	90.00%	90.00%	80.00%	100.00%	85.00%	95.00%	90.00%	90.00%
Contact-GraspNet	100.00%	90.00%	90.00%	90.00%	95.00%	85.00%	100.00%	85.00%	95.00%	95.00%	92.50%
Ours	100.00%	90.00%	90.00%	90.00%	95.00%	90.00%	100.00%	90.00%	95.00%	95.00%	93.50%

**Table 6:** Experimental results of multi-object grasping.

Method	Success Rate	Completion Rate	Time Efficiency
GPD (3 channels)	66.00%	82.00%	22,697 ms
GPD (12 channels)	71.00%	89.00%	25,712 ms
S^4^G	78.00%	91.00%	8159 ms
Contact-GraspNet	81.00%	94.00%	12,861 ms
Ours	81.00%	95.00%	13,296 ms
